# 1401. Asymptomatic Visceral Leishmaniasis in U.S. Soldiers Previously Deployed to Afghanistan

**DOI:** 10.1093/ofid/ofad500.1238

**Published:** 2023-11-27

**Authors:** Michael Stein, Hui Liu, Dutchabong Shaw, Fernanda Fortes de Araújo, Nancy L Koles, David Saunders, Naomi E Aronson, Naomi E Aronson

**Affiliations:** USU, Tacoma, Washington; USUHS, Bethesda, Maryland; HJF/USU, Bethesda, Maryland; Uniformed Services University of the Health Sciences, Bethesda, Maryland; HJF/USUHS, Severn, Maryland; Uniformed Services University of the Health Sciences, Bethesda, MD, USA, Bethesda, Maryland; Uniformed Services University of the Health Sciences, Bethesda, Maryland; Uniformed Services University of the Health Sciences, Bethesda, Maryland

## Abstract

**Background:**

Visceral leishmaniasis (VL) is a chronic protozoal disease caused from the bite of an infected sand fly. VL due to *Leishmania infantum* is endemic in Afghanistan, although its distribution is poorly characterized. The spectrum of VL ranges from asymptomatic to active VL with symptoms of fever, weight loss, organomegaly, and pancytopenia. Most symptomatic patients die without treatment; asymptomatic patients remain at risk lifelong for symptomatic reactivation—especially if immunosuppressed. Travelers returning from VL endemic areas may harbor latent infection years after their return. We aimed to determine the prevalence of asymptomatic VL (AVL) in US military personnel previously deployed to Afghanistan.

**Methods:**

Healthy adult US military personnel who deployed to Afghanistan over summer months were recruited from the Washington DC area. 90 volunteers completed a risk factor survey, blood draw, and had completed results. Diagnostic testing utilized *Leishmania*

ELISA, interferon gamma release assay (IGRA), and quantitative PCR (qPCR). Statistical analyses included Fisher exact test, Pearson χ2 test, Welch Two Sample t-test, and Mann-Whitney U test. IRB approval was obtained.

**Results:**

The prevalence of AVL (ELISA, IGRA, or qPCR positivity) in the volunteers was 9/90 (10%). Two (2.2%) PCR, 7 (7.8%) ELISA, and no (0%) IGRA samples were positive. AVL+ participants were a median of 10.6 years (range 8 - 18.6) post Afghanistan deployment, and compared to AVL- volunteers, were older (median 50 versus 40 years, p= 0.008) and more likely to self-identify as African-American (n = 5 (56%) vs 16 (20%), p = 0.029). No risk factors for AVL were identified in exploratory analysis of the volunteers’ military roles, types of outdoor exposure, or deployment location within Afghanistan.

**Conclusion:**

In this preliminary cross-sectional analysis of US military personnel who returned from Afghanistan, the prevalence of AVL was 10%–comparable to other published studies on the prevalence of AVL in endemic areas and of concern when one considers the several million US military personnel previously deployed to Iraq and Afghanistan. Due to the persistence of this intracellular infection, clinicians should be alerted for reactivation potential in previously deployed Servicemembers.

**Disclosures:**

**Naomi E. Aronson, MD**, british medical journal: Honoraria|British Medical Journal: honoraria for writing chapter for Best Evidence|Elsevier: royallties serve as textbook editor|Elsevier: Royalties as text editor|UpTo Date: royalties for writing chapters|UpToDate: royalties for writing chapters|Wellcome Foundation: Honoraria|Wellcome Foundation: program advisory board|Wellcome Trust: Honoraria|Wellcome Trust: program advisory board **Naomi E. Aronson, MD**, british medical journal: Honoraria|British Medical Journal: honoraria for writing chapter for Best Evidence|Elsevier: royallties serve as textbook editor|Elsevier: Royalties as text editor|UpTo Date: royalties for writing chapters|UpToDate: royalties for writing chapters|Wellcome Foundation: Honoraria|Wellcome Foundation: program advisory board|Wellcome Trust: Honoraria|Wellcome Trust: program advisory board

